# Organ-specific expression of genes involved in iron homeostasis in wheat mutant lines with increased grain iron and zinc content

**DOI:** 10.7717/peerj.13515

**Published:** 2022-06-10

**Authors:** Saule Kenzhebayeva, Saule Atabayeva, Fatma Sarsu, Alfiya Abekova, Sabina Shoinbekova, Nargul Omirbekova, Gulina Doktyrbay, Aizhan Beisenova, Yuri Shavrukov

**Affiliations:** 1 Department of Biotechnology/Faculty of Biology and Biotechnology, Al-Farabi Kazakh National University, Almaty, Kazakhstan; 2 Department of Biotechnology/Faculty of Biology and Biotechnology, Al-Farabi Kazakh National University, Almaty, Kazakhstan; 3 Plant Breeding and Genetics Section, General Directorate of Agricultural Research and Policies, Ankara, Turkey; 4 Kazakh Research Institute of Agriculture and Plant Growing, Almaty Region, Kazakhstan; 5 Department of Biotechnology/Faculty of Biology and Biotechnology, Al-Farabi Kazakh National University, Almaty, Kazakhstan; 6 Department of Molecular Biology, Asfendiyarov Kazakh National Medical University, Almaty, Kazakhstan; 7 College of Science and Engineering (Biological Sciences), Flinders University of South Australia, Adelaide, Australia

**Keywords:** Organ-specific gene expression involved in iron homeostasis, Iron bioavailability and homeostasis, Spring wheat mutant lines, Fe regulation and storage in grains

## Abstract

**Background:**

Iron deficiency is a well-known nutritional disorder, and the imbalance of trace-elements, specifically iron, is the most common nutrient deficiency of foods across the world, including in Kazakhstan. Wheat has significant nutritional relevance, especially in the provision of iron, however many bread wheat varieties have low iron despite the need for human nourishment. In this study, the expression profiles of wheat homologous genes related to iron homeostasis were investigated. The work resulted in the development of two new M_5_ mutant lines of spring bread wheat through gamma-irradiation (200 Gy) with higher grain iron and zinc content, lower phytic acid content, and enhanced iron bioavailability compared to the parent variety. Mutant lines were also characterized by higher means of yield associated traits such as grain number per main spike, grain weight per main spike, grain weight per plant, and thousand-grain weight.

**Methods:**

The homologous genes of bread wheat from several groups were selected for gene expression studies exploring the tight control of iron uptake, translocation rate and accumulation in leaves and roots, and comprised the following: (1) S-adenosylmethionine synthase (*SAMS*), nicotianamine synthase (*NAS1*), nicotianamine aminotransferase (*NAAT*), deoxymugineic acid synthetase (*DMAS*), involved in the synthesis and release of phytosiderophores; (2) transcription factor basic helix-loop-helix (*bHLH*); (3) transporters of mugineic acid (*TOM*), involved in long-distance iron transport; (4) yellow stripe-like (*YSlA*), and the vacuolar transporter (*VIT2*), involved in intracellular iron transport and storage; and lastly (5) natural resistance-associated macrophage protein (*NRAMP*) and ferritin (*Fer1A*).

**Results:**

The wheat homologous genes *TaSAMS*, *TaNAS1*, and *TaDMAS*, were significantly up-regulated in the roots of both mutant lines by 2.1–4.7-fold compared to the parent variety. The combined over-expression of *TaYSlA* and *TaVIT2* was also revealed in the roots of mutant lines by 1.3–2.7-fold. In one of the mutant lines, genes encoding intracellular iron transport and storage genes *TaNRAMP* and *TaFer1A-D* showed significant up-regulation in roots and leaves (by 1.4- and 3.5-fold, respectively). The highest expression was recorded in the transcription factor *TabHLH*, which was expressed 13.1- and 30.2-fold in the roots of mutant lines. Our research revealed that genotype-dependent and organ-specific gene expression profiles can provide new insights into iron uptake, translocation rate, storage, and regulation in wheat which aid the prioritization of gene targets for iron biofortification and bioavailability.

## Introduction

Iron (Fe) is an essential micronutrient for plant growth, development, productivity, and product quality (Fageria et al., 2012). It is also required for the proper functioning of a broad spectrum of metabolic reactions in humans (Lieu et al., 2001). A nutritious human ration comprises 43–78% of cereals and legumes, characteristic of people in developing countries (Black, Moon & Baird, 2014). Bread wheat (*Triticum aestivum* L.) is a staple crop that is also a daily source of nutrients. Wheat grain has a low Fe/Zn level, which is the reason for widespread Fe deficiency in populations with wheat dominant in the diet (Balk et al., 2019; Ali & Borrill, 2020). Among them, Fe deficiency is the most common nutrient shortage for more than three billion people worldwide (https://ourworldindata.org/micronutrient-deficiency). It should be noted that a decrease in micronutrient content including Fe and Zn in bread wheat grain may be intensified by the ubiquitous application of nitrogen fertilizers and long-term tillage, leading to a reduction in their bioavailability to plants (Shiwakoti et al., 2019). It was reported that a decline in Fe and Zn content was found in wheat grain with high N fertilization rates (Cakmak, Pfeiffer & McClafferty, 2010). Therefore, increasing Fe content in wheat grain is a vital nutritional and economically important goal.

As documented, the Fe level in grain depends on the closely controlled absorption, transport and storage of Fe, which are complex processes involving many genes (Connorton et al., 2017; Wu & Ling, 2019). Plants have developed various strategies to absorb adequate soil Fe. All dicots and non-graminaceous monocots utilize the rhizosphere acidification (Strategy I) mechanism where metal uptake is provided by the release of H^+^ *via* proton-root plasma membrane H^+^-ATPases that increase Fe^3+^ solubility (Ali & Borrill, 2020; Gao & Dubos, 2021; Gupta et al., 2021). In Strategy II, a chelation-based strategy enhances Fe uptake acquisition from soil in the oxidized ferric state, representing the dominant mechanism in cereals. This strategy involves the utilization of a variety of phytosiderophores (PSs) such as nicotinamine (NA), 2′-deoxymugineic acid (DMA), and mugineic acids (MAs). These are effluxed from the plant roots into the rhizosphere and chelate inorganic insoluble ferric Fe (Fe (III)) to form Fe^3+^-PS complexes and contribute to the absorption of Fe (Curie et al., 2001; Kobayashi & Nishizawa, 2012; Paul et al., 2013; Dey et al., 2020; Gupta et al., 2021). In gramineous plants, S-adenosyl methionine (SAM), the methionine-activated form, participates in the biosynthesis of MAs and plays a key role in Fe uptake through NA metabolism, which is a general Fe chelator (Bashir et al., 2006; Grillet & Schmidt, 2019). Nicotianamine synthase (NAS) catalyzes the synthesis of NA from SAM and activates genes encoding enzymes that are differentially regulated by Fe-status in bread wheat and may function in multiple processes due to fact that NA is implicated in Fe uptake, transport/translocation and homeostasis (Bonneau et al., 2016; Shi, Li & Sun, 2020). The NA biosynthesis involves only one enzymatic step, mediated by nicotianamine synthase (NAS), using SAM as a substrate. Increased levels of NA also led to higher grain Zn and manganese content, since NA supports the mobilization of these bivalent metals, among others (Curie et al., 2009; Clemens et al., 2013). The Fe^3+^-PS complexes are transported across membranes by YSL transporters, such as YSL2 in rice (Ishimaru et al., 2010). The role of *YSL15* has been confirmed in rice (Inoue et al., 2009; Lee et al., 2009; Kumar et al., 2019). In wheat also, 67 *YSL* genes have been identified using transcriptome analysis (Kaur et al., 2019). YSLs proteins, which also function in shoots but to a lesser degree than in roots, promote long-distance transport from leaves to grain through both xylem and phloem (Kenzhebayeva, Atabayeva & Sarsu, 2021). Biofortification attempts have focused on NA, as it is specifically involved in the transport of divalent metals and not, like citrate, a more general metabolite (Connorton & Balk, 2019).

Nicotianamine aminotransferase (NAAT) and deoxymugineic acid synthase (DMAS) catalyze the formation of DMA from NA during MA biosynthesis (Takahashi et al., 2003; Bashir et al., 2006; Bonneau et al., 2016). The reaction of NA transamination with NAAT occurs only in gramineous plants, which is evidence of the first step of Strategy II (Takahashi et al., 2003; Dey et al., 2020). Bread wheat is characterized by the secretion of only one type of MA, such as DMA (Ma et al., 1999). As has been reported in wheat, the genes responding to Fe uptake involve *TaNAAT*, *TaDMAS*, *TaYSL*, *TaZIFL* and some others (Beasley, Bonneau & Johnson, 2017; Kumar et al., 2019; Sharma et al., 2019). Wherein zinc-induced facilitator-like (ZIFL) transporters engage in the release of PSs to enhance Fe/Zn uptake *via TaZIFL* (Sharma et al., 2019). The differential and gradual upregulation of *TaNAAT1*, *TaNAAT2*, and *TaDMAS1*, encoding the corresponding enzymes, was observed in the root tissues of bread wheat after a short-term Fe deficit (Beasley, Bonneau & Johnson, 2017).

The efflux of PSs forming the Zn^3+^/Fe^3+^-PS complexes and the acquisition of chelated metal ions are reported to be provided by several transporters, including TOM (transporter of MA) and yellow-stripe-like proteins (YSLs), and the corresponding genes encoding these transporters have been recently studied (Kumar et al., 2019; Wang, Kawakami & Bhullar, 2019; Gupta et al., 2021). In rice and barley, efflux transporters (ETs) such as transporter of mugineic acid 1 (*OsTOM1*), *OsTOM2* and *HvTOM1* bind to DMA and export it from the root to the soil. DMA chelates soil Fe (III) and is further absorbed as the DMA-Fe (III) complex through the Yellow Stripe-Like (YSL) type of Influx Transporters (ITs) present in the root (Nozoye et al., 2011; Paul et al., 2013). In these plants, TOM1 helps to transport Fe (III) in the DMA-chelated form into the xylem and the phloem (Nozoye et al., 2011). The plant shoot YSL transporters could also contribute to the long-distance transport of Zn^3+^/Fe^3+^-PS complexes from leaves to grains through both xylem and phloem. The biotic and abiotic stresses that induce differential expressions of wheat YSLs underline their various functions in growth processes. YSL transporters could be considered a significant target in prioritizing genes to address wheat biofortification, which is the key to preventing human malnutrition (Kumar et al., 2019). Our study revealed that at Fe limited growth conditions, levels of *TaYS1A* showed 2.6–5.9-fold increases in the roots but not in shoots, with high-level expression (15.3–60.8 times compare to in shoots) in Almaken and Zhenis M_5_ mutant lines, the new Fe-biofortified spring wheat genotypes (Kenzhebayeva, Atabayeva & Sarsu, 2021). Such tissue-specific activity of the transporter could be suggested as an appropriate strategy for Fe loading into grains without disturbing the morphology of the plants. In the symplastic route of metal ions for radial transport to the shoot, the natural resistance-associated macrophage protein (NRAMP) transporter is essential. The corresponding genes for NRAMPs described in dicots have also been identified in rice, barley, maize, and wheat (Ali & Borrill, 2020; Gupta et al., 2021). In addition, genes including *TaNAM*, *TaGPC* and *TaVIT* directly or indirectly regulate Fe and Zn transportation and/or translocation from vegetative tissues to wheat grain (Uauy et al., 2006; Waters et al., 2009; Pearce et al., 2014; Connorton et al., 2017).

Several transcription factors (TFs), such as those from the bHLH family, are involved in the regulation of Fe homeostasis (Heim et al., 2003; Gao et al., 2019; Wu & Ling, 2019; Hao et al., 2021). In wheat, tissue- and growth stage-specific regulation of *TabHLH* has been reported (Wang, Kawakami & Bhullar, 2019). Under low Fe growth conditions, Fe ion absorption and transport appear to be guided by the Fe deficiency-inducible bHLH family TFs of both in shoots and roots but with a much higher level in shoots (Kenzhebayeva, Atabayeva & Sarsu, 2021). In wheat, *TabHLH1*, one such member of this type of TF gene, improved plant tolerance to phosphorus and nitrogen deprivation by regulating nutrient transporter gene transcription and reactive oxygen species homeostasis (Yang et al., 2016).

Within the grain, ferritin complexes in the case of Fe and the vacuole for both Zn and Fe are two important locations for the deposition of micronutrients (Gupta et al., 2021). Plant ferritin is a ubiquitous protein whose synthesis is controlled by Fe status (Briat et al., 2010; Borg et al., 2012; Connorton & Balk, 2019). Ferritin plays a significant role in cereals, functioning as the storage protein under Fe in excess condition, and during metal shortage, it functions to release Fe (Briat et al., 2010). In the Fe homeostasis of cereals plants, Fe in a free state can cause the formation of free radicals, thus leading to plant damage. It has also been shown that despite the presence of ferritin for the storage of Fe, only a very small portion of the total Fe is stored *via* ferritin; most of the remaining metal is stored in vacuoles (Gupta et al., 2021). Induction of ferritin transcript levels was observed under Fe abundance, which indicates its importance in plant Fe homeostasis and the ability to adaptively respond to environmental conditions (Briat et al., 2010). Regarding *Fer1A-D*, in our previous study, for wheat Almaken mutant lines, transcript levels were greatly down-regulated under Fe starvation in shoots. There were no differences at Fe-normal and Fe-deficient growth conditions indicating the relation of genotype-dependent and tissue-specific *Fer1A-D* gene expression (Kenzhebayeva, Atabayeva & Sarsu, 2021).

Wheat grains have relatively low essential microelements, especially Fe and Zn, which tend to decrease as the yield increases (White & Broadley, 2005; Fan et al., 2008; https://www.who.int/nutrition/topicsida/en). Among the many ways of increasing Fe content, such as through conventional breeding and transgenic methods, biofortification of staple food crops through breeding is a key strategy for decreasing human mineral deficiency and one of the most cost-effective, sustainable, and environmentally safe approaches for the prevention and alleviation of nutrient malnutrition in humans (Fageria et al., 2012; Borrill et al., 2014; Balk et al., 2019; Gupta et al., 2021).

Genetic improvement of modern cereal cultivars through breeding efforts focused on biofortification presents a long-term approach to the global problem of micronutrient malnutrition. Induced mutagenesis is one of the most powerful tools for extending the genetic variation demanded in developing valuable germplasm sources to meet the needs of sustainable agriculture within the scope of climate change (Parry et al., 2009; Chaudhary, Deshmuk & Sonah, 2019; Viana et al., 2019; Ahumada-Flores et al., 2021). New cereal mutant germplasm created through mutation selection can improve deployment and application, incorporate positive qualities into new cultivars, or create new mutant varieties for better sustainable agriculture. Mutagenesis has been widely used to increase yields, but less so to improve nutritional value (Parry et al., 2009). Notably, mutant varieties are not recognized as genetically modified organisms and are distributed widely across all countries without public skepticism or additional regulatory limitations. Achievements in molecular biology have greatly advanced mutational breeding, making it more expedient than previously considered (Chaudhary, Deshmuk & Sonah, 2019).

Current knowledge is not sufficient to determine how wheat genotypes regulate the specific genes involved in Fe homeostasis. There are no published reports of any strong association between increased grain Fe and Zn content of mutant spring bread wheat lines and differential expression of genes involved in iron homeostasis in roots and leaves.

The present study focused on investigating the organ-specific expression of genes related to MA synthesis and secretion, long-distance Fe transport, intracellular Fe transport and storage, and transcriptional regulation, associated with a variation in grain Fe and Zn content. Two new mutant lines (M_5_ generation), based on spring wheat cv. Erythrospermum-35 (wild type, WT), were selected as the experimental material (Kenzhebayeva et al., 2017). These lines were derived through 200 Gy gamma treatment to widen genetic variation and obtain significantly higher grain Fe and Zn content, lower phytic acid (PA) content, enhanced metal bioavailability and higher main components of productivity compared to the WT parent. cv. Erythrospermum-35. The differential expression of genes investigated in the roots and leaves indicated genotype- and tissue-specific roles for Fe chelators, transporters, and transcription factors, appending new insights into genes involved in Fe homeostasis and identifying new sources for wheat biofortification.

## Materials and Methods

### Plant material

Grains of the spring bread wheat WT cv. Erythrospermum-35 (*Triticum aestivum* L.) were received from the Kazakh Research Institute of Agriculture and Plant Growing (KLRIAPG), Almaty region, Kazakhstan, and 2,000 grains were irradiated with 200 Gy doses from a ^60^Co source at the Kazakh Nuclear Centre, Almaty, Kazakhstan following the described method (Kenzhebayeva et al., 2019). After irradiation, the grains were germinated and sown to raise the in an experimental field of KRIAPG (43°15 N, 76°54 E, 550 m) to raise M_1_ plants. Single spikes from each plant from the M_2_ generation were harvested from 150 plants for analysis, and the 60 best lines were selected based on the yield of individual plants. This process was repeated until the M_5_ generation using the single main spike strategy. Although the number of plant tillers and spikes varied, the seeds were collected from a single main spike. The best mutant lines were selected individually according to the grain weight of the main spike (GWS) and grain weight per plant (GWP). These selection criteria were applied in the M_3_ and M_4_ generations based on the values for the WT parent, cv. Erythrospermum-35, grown under the same conditions. Grains from each mutant line and WT parent were planted in a trial for further evaluation. In 2011, WT cv. Erythrospermum-35 had a mean GWS of 1.15 ± 0.40 g and a mean GWP of 1.36 ± 0.21 g. In 2012, for the M4 generation, the threshold criteria chosen for selection were GWS > 1.8 g and GWP > 2.5 g for the mutant lines. The mutant lines were grown as three-replicate three-row plots, 2 m long and 1.2 m wide, with 20 cm between rows and 30 seeds per row. The experiment was conducted according to locally recommended agronomic practices. The types of fertilizer application, time of use, and soil conditions were as described earlier (Kenzhebayeva et al., 2017). No additional fertilizer containing Fe, Zn, or other metals was used. Ten mutant lines were randomly selected for advanced analysis (five samples per row).

Two M_5_ mutant lines were evaluated on yield associated traits (Table S1). These were identified as genotypes with significantly higher grain Fe and Zn content, lower phytic acid content, and improved Fe/Zn bioavailability: M/1 (144/1) and M/2 (153/5), both with the background of WT cv. Erythrospermum-35 (Table 1). The mutant lines were selected for molecular analysis to understand the role of metal homeostasis-related genes in Fe/Zn uptake, transport, and redistribution in the leaves and roots of mutant lines alongside the parent variety.

**Table 1:** Grain content of Fe, Zn, phytic acid (PA) and protein (GPC), grain morphological traits including grain length (GL), grain width (GW) and grain area (GA), in WT parent, spring bread wheat cv. Erythrospermum-35 and two M_5_ mutant lines.

Grain quality trait	WT, cv. Erythrospermum-35	Mutant line M/1	Mutant line M/2
Grain Fe content, mg/kg	35.36 ± 0.71	56.25 ± 2.51**	60.64 ± 0.86**
Grain Zn content, mg/kg	29.35 ± 2.68	46.91 ± 2.25**	61.03 ± 1.23**
Grain PA content, mg/g	7.40 ± 0.68	4.10 ± 0.88*	2.64 ± 0.50**
РA:Fe molar ratio	17.70	6.11*	3.68*
РA:Zn molar ratio	24.97	8.66*	4.28*
GPC, %	13.3 ± 0.1	13.03 ± 0.06	13.67 ± 0.15*
GL, mm	7.28 ± 0.31	8.67 ± 0.15*	8.31 ± 0.89
GW, mm	3.55 ± 0.8	3.67 ± 0.61	3.65 ± 0.64
GA, mm^2^	17.45 ± 0.22	19.65 ± 0.22**	19.82 ± 0.18**

**Notes:**

Data presented as mean for three extracts and analysis repetitions ± standard deviation.

Asterisks indicate the statistical difference between the WT parent and the mutant lines (*P < 0.05; and **P < 0.01).

### Measurement of yield associated traits in WT spring wheat cultivar and two M_5_ mutant lines

To record yield associated traits, the plant parameters were measured as follows: grain number per main spike (GNS), grain weight per plant (GWP) and grain weight per main spike (GWS) using a standard laboratory balance with two decimal places. The thousand grain weight (TGW) was calculated as the mean weight of three sets of 100 grains per line multiplied by 10.

### Plant growth conditions

Grains of two mutant lines, M/1 and M/2, and the WT parent were germinated on wet filter paper for 7 days. Seedlings were transferred to the hydroponic system under greenhouse conditions (22 °C/18 °C with a 16-h light/8-h dark cycle and 60% humidity). The plants were grown in solutions (pH 6.0) with sufficient Fe including, 0.88 mM K_2_SO_4_, 2 mM Ca(NO_3_)_2_, 0.2 mM KH_2_PO_4_, 1.0 mM MgSO_4_, 0.1 mM KCl, 1.0 μM H_3_BO_3_, 1.0 μM MnSO_4_, 0.2 μM CuSO_4_, 0.02 μM (NH_4_)_6_Mo_7_O_24_, 1.0 μM ZnSO_4_, and 100.0 μM F (III)-EDTA. Plants were grown under the following conditions: parent, WT, cv. Erythrospermum-35 and two mutant lines × condition (sufficient Fe) × three biological replicates. The hydroponic solution was replaced every 7 days, and the solution pH was adjusted twice per week. Air was continuously pumped into the hydroponic solution to improve circulation. Roots and leaves were sampled for analysis 42 days after transplantation into the hydroponic solution. Three biological replicates were collected for each sample.

### Perls’ Prussian blue grain Fe staining

After incubation at 30 °C for 40 min, slightly swollen mature grains were horizontally and longitudinally dissected using a platinum-coated scalpel, stained for 60 min in Perls’ Prussian blue staining solution (2% [w/v] potassium hexacyanoferrate [II] and 2% [v/v] hydrochloric acid), and then washed twice in deionized water as described by Connorton et al. (2017).

### Dithizonate grain Zn staining

To study the localization of grain Zn, a staining method was developed using dithizonate (DTZ, diphenyl thiocarbazone), which produces a red to purple coloured Zn-dithizonate complex (McNary, 1954). Similar as above, slightly swollen mature grains were horizontally and longitudinally dissected using a platinum-coated scalpel, stained for 60 min in DTZ staining solution (0.05% w/v DTZ in 99.8% trichloromethane), and then rinsed thoroughly in water.

### Assessment Fe and Zn content in grain and roots

Grain samples of two M_5_ mutant lines and the WT parent, Erythrospermum-35, were washed with sodium dodecyl sulfate (0.1%), rinsed in deionized water, dried to a constant weight at 65–70 °C, and then ground using a mixer mill (Retsch MM400; Retsch, Haan, Germany). Sample digestion (0.2 g) and subsequent extraction was carried out as described previously (Kenzhebayeva et al., 2017). The grain Fe and Zn contents were measured using flame atomic absorption spectroscopy (Model NovAA350; Analytik Jena, Jena, Germany). Metal measurements were checked against the certified reference values from the national standard samples LLC ‘HromLab’, Fe 7835-2000 and Zn-7256-96 diluted with 0.3% HNO_3_. To measure Fe content in roots, the plants were grown in similar conditions with nutritional solutions as described above but in a separate experiment. The Fe translocation factor (rate) from the root to grain (TF_root/grain_) was measured as follows: TF_root/grain_ = C_grain_/C_root_, where *C*_grain_ was Fe content in grains (mg/kg) and *C*_root_ was Fe content in roots (mg/kg). Three extracts and repetitions were performed for analysis.

### Phytic acid extraction and determination, and calculation of the PA:Fe and PA:Zn molar ratios

The extraction of PA from milled grain samples (0.3 g) was performed with 7 mL 0.66 M HCl stirred vigorously overnight at 22 °C following the manufacturer instructions provided with the Megazyme quantitative method kit K-PHYT 05/17. PA was determined by enzymatic treatment with a phytase that is specific for PA, and subsequent treatment with alkaline phosphatase ensures the release of the final phosphate from myo-inositol phosphate (IP1). The total phosphate released was measured using a modified colorimetric method with the following conversion to PA content, as described by the Megazyme kit. To calculate the molar ratios of РA:Fe and PA:Zn, the contents of РA and the metals were converted into moles by dividing by their respective molar masses and atomic weights.

### Grain morphology and protein content analysis

Measurements of grain morphology were made using the WinRHIZO image analysis system (version 1.38 2007; Reagent Instruments Inc., Quebec, Canada), and included: grain length (GL), grain width (GW) and grain area (GA) on 50 grains per genotype.

Grain protein content was determined in whole grains using near-infra red reflectance spectroscopy (ZX50 Portable Grain Analyzer, Vernon Hills, IL, USA) with proprietary calibration software provided (Zeltex Hagerstown, Hagerstown, MD, USA). Three repetitions were performed using 25 grains per genotype.

### RNA extraction, cDNA synthesis and gene expression analysis

Total RNA was extracted from roots and leaves of 42-day old plants using the Isol-RNA lysis reagent (5 PRIME GmbH, Hamburg, Germany). The RNA integrity and quality was checked by running 1 µL of the extracted RNA in a 1% agarose gel. Each RNA sample (2 µg) was treated with 1 µL of DNAse I (Life Technologies, Thermo Fisher Scientific, Vilnius, Lithuania) followed by cDNA synthesis in a 20 µL volume reaction using the RevertAid First Strand cDNA Synthesis Kit (Life Technologies, Thermo Fisher Scientific, Vilnius, Lithuania). All procedures were carried out according to the manufacturers’ instructions.

Samples of cDNA diluted with water (1:10) were used for qPCR analyses with a LightCycler 480 Instrument II Real Time PCR system (Roche, Basel, Switzerland) following the qPCR protocol described earlier (Tejada-Jiménez et al., 2015). The total volume (10 μL) in each well included 5 μL of SYBR Green PCR Master Mix (Applied Biosystems, Waltham, MA, USA), 3 μL of diluted cDNA, and 1 μL of 3 μM each F and R gene-specific primers (Table S2), as per the manufacturer’s recommendation. Thermal cycling conditions included incubation at 50 °C for 2 min, initialization at 95 °C for 10 min, followed by denaturation at 95 °C for 15 s, and annealing and extension at 60 °C for 1 min, which were repeated for 40 cycles. Expression data for the target genes were normalized relative to the expression of the reference gene Ta.22845, *ATP-dependent 26S proteasome regulatory subunit* (Paolacci et al., 2009), as one of the most stable reference gene across all of the tested samples. Sequences of all used primers for 10 studied genes and reference gene with amplicon sizes and primer efficiency are also presented in Table S2. Primer efficiency was determined using a standard curve calculated with the following formula: E = 10^(−1/slope)^. Melting curves analysis was performed after qPCR cycling with an intercalating dye had a single distinct peak. Three biological and two technical replicates were used in each qPCR experiment. Data normalization was performed as described earlier (Schefe et al., 2006).

### Statistical analysis

Statistical analysis including one-way ANOVA, SD, SEM, and *P*-value, was carried out using R-Studio (Version 1.1.456) and Excel software. All values are expressed as the mean of three measurements (biological replicates) for each gene. Correlation coefficients (r^2^) between productivity components, grain morphometry, and grain nutritional characteristics (Fe, Zn and PA content), and *P* values were calculated using the GenStat software (10^th^ edition) (Table S3).

## Results

### Accumulation of Fe, Zn, PA, grain protein and grain morphometry analysis in two M_5_ mutant lines compared to WT

**Figure 1:**
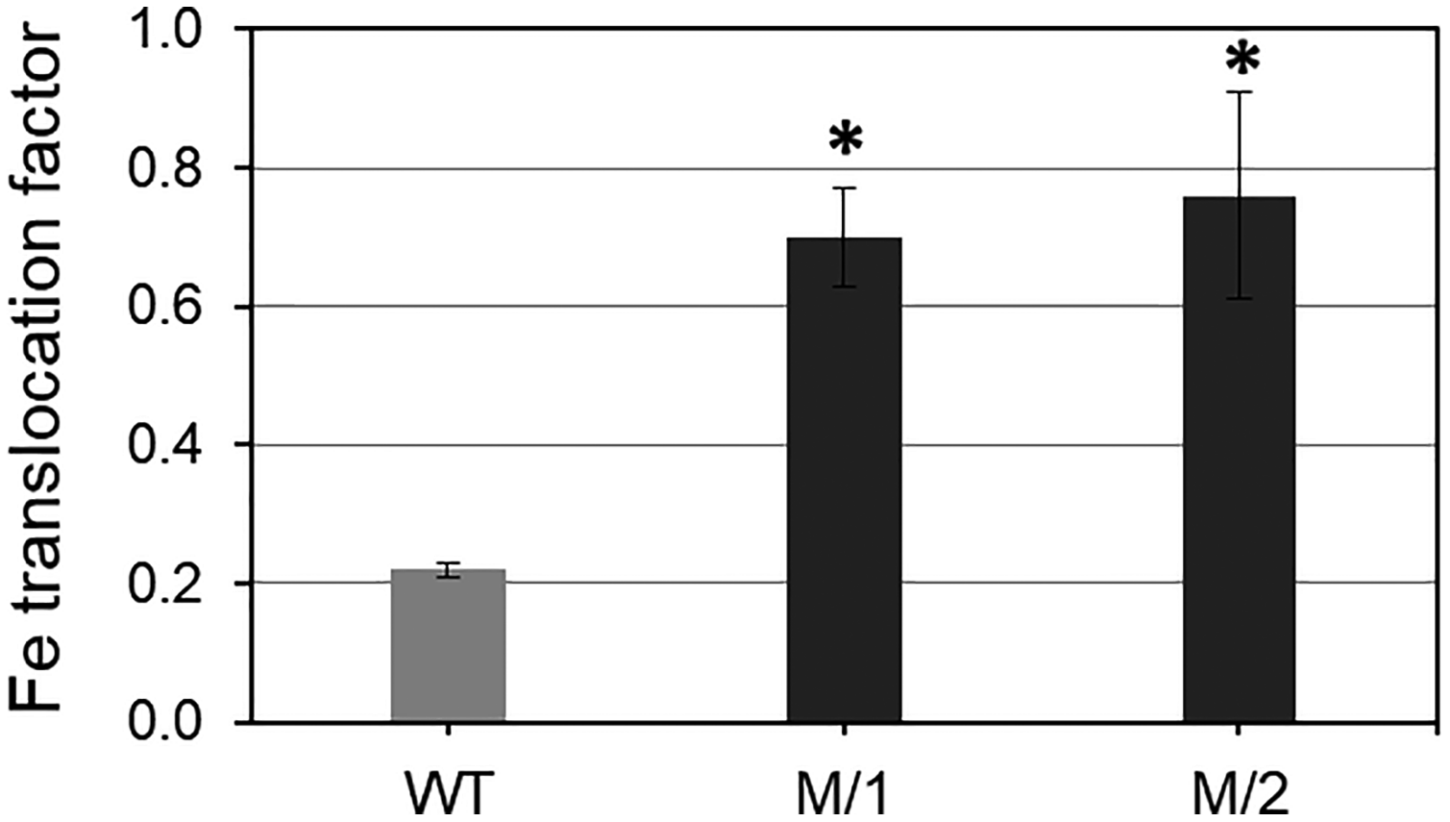
Translocation factor (rate) of Fe from roots to mature grains of spring bread wheat cv. Erythrospermum-35 and two M5 mutant lines, M/1 and M/2. To measure Fe content in roots, the plants were grown in nutritional solutions. Values are the mean ± SD deviation from the three biological replicates. Asterisks indicate significant differences between WT parent and the mutant lines (*P < 0.05).

Two mutant lines (M/1 and M/2) were selected from 32 lines of the M_5_ generation after 200 Gy gamma-radiation treatments on the background of the original parent, WT spring bread wheat cv. Erythrospermum-35. The comparison of the M/1 and M/2 mutant lines with the WT revealed a significantly increased Fe and Zn content in grains and, at the same time, significantly decreased PA content (Table 1). The mutant lines had 1.6- and 1.7-fold more grain Fe content and 1.6–2.1-fold more Zn content in grains compared to the original WT cultivar. Importantly, PA accumulation in grain of the mutant lines was 1.8- and 2.8-fold less than in WT plants. These results indicate that the bioavailability of important metals Fe and Zn were 2.9–5.8 times higher than in the WT parent wheat cv. Erythrospermum-35, which strongly improves metal bioavailability in grains of both studied mutant lines (Table 1). In the current study, the Fe translocation factor from roots to grains was significantly higher in both mutant lines compare to WT, cv. Erythrospermum-35 (Fig. 1).

Our presented results revealed that grain protein content (GPC) was significantly higher (10.3%) in one mutant line M/2 compared to the WT parent (Table 1).

In the present study, there was significantly increased grain length (GL), and area (GA) in one and both mutant lines, respectively, but not in grain width (GW) compared to the WT parent (Table 1).

### The distribution of Fe and Zn in grain of WT and two M_5_ mutant lines

**Figure 2:**
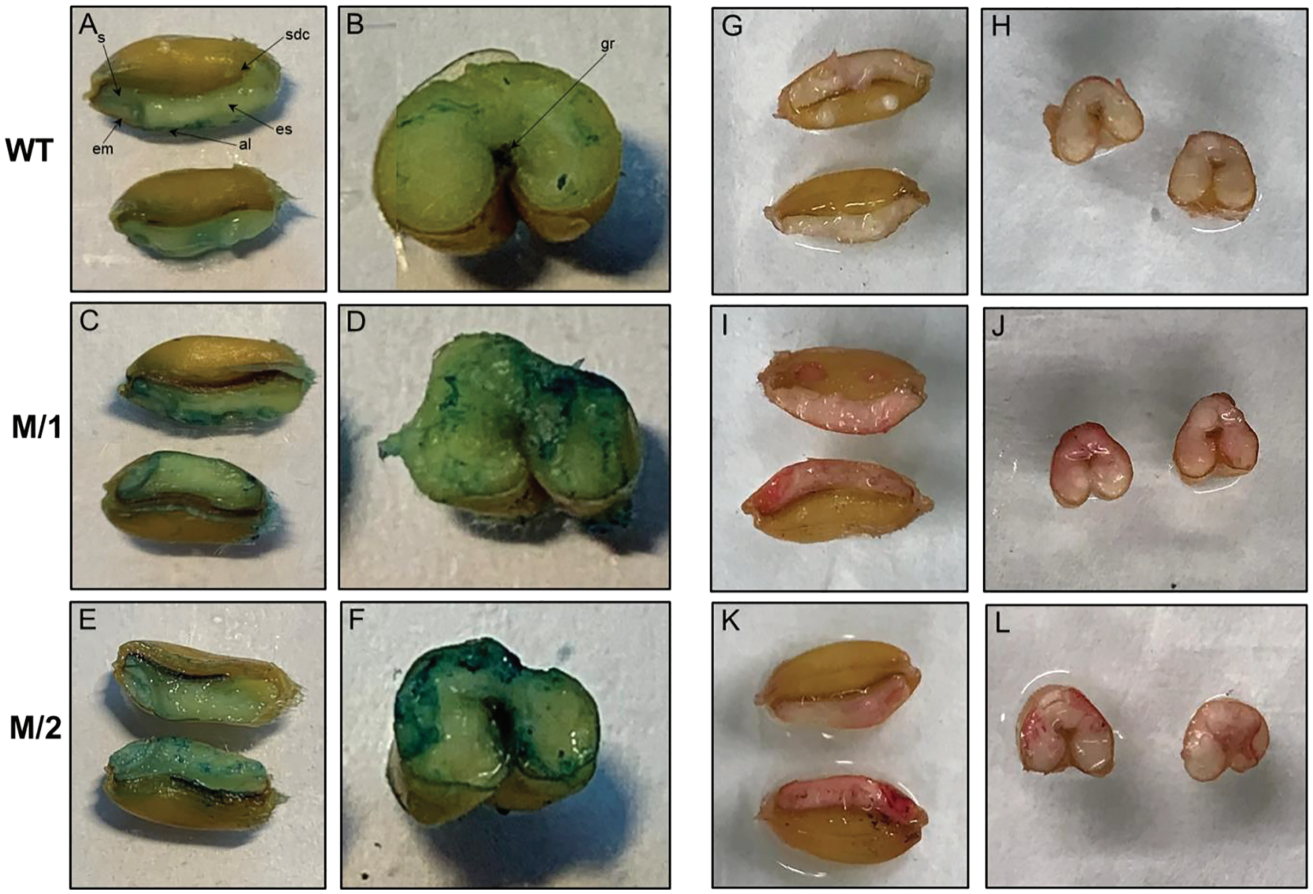
Perls’ Prussian blue staining for Fe is present in panels A–F, and Dithizonate staining for Zn is present in panels G–L. Iron staining in grains of WT, cv. Erythrospermum-35 (A and B), mutant lines M/1 (C and D), and M/2 (E and F), where seeds were dissected longitudinally (A, C and E) and transversely (B, D and E). Abbreviations inside of the Figures: al, aleurone; em, embryo; es, endosperm; gr, groove; s, scutellum; sdc, seed coat. Zinc staining in grains of WT, cv. Erythrospermum-35 (G and H), mutant lines M/1 (I and J), and M/2 (K and L), where seeds were dissected longitudinally (G, I and K) and transversely (H, J and L).

Horizontally and longitudinally dissected mature wheat grains from WT cv. Erythrospermum-35, and two mutant lines M/1 and M/2 were Fe stained using Perls’ Prussian blue (Figs. 2A–2F). In WT, positive blue staining was visible around the embryo, aleurone layer and slightly in scutellum but the endosperm had very little Fe. In mutant lines with higher content of Fe than cv. Erythrospermum-35 (Table 1), the Perls’ Prussian blue staining was noticeably increased and was distributed in the embryo, scutellum, most prominent in the aleurone layer, and, in contrast to WT, particularly strong around the groove and in patches of endosperm (Figs. 2C–2F).

The localization of Zn was detected in grains horizontally and longitudinally disserted for Zn staining with DTZ. When staining for Zn, DTZ forms a pink or red coloured Zn-DTZ complex (Figs. 2G–2L). The formation of the Zn-DTZ complex in grains of both mutant lines was more intense with higher Zn content compared to the WT. Genotypic differential localization of Zn in the grains was more apparent in the longitudinal sections of seeds. In contrast to cv. Erythrospermum-35, the DTZ staining revealed that the Zn-DTZ complex was more intense in the embryo, aleurone layer and endosperm (Figs. 2I–2L).

### Evaluation of yield-associated traits in WT and two M_5_ mutant lines

For new wheat germplasm, yield is unalterably the essential index. Therefore, analysis of yield associated traits was carried out including GNS, GWS, GWP, and TGW. The results for these parameters showed that mutant lines significantly exceed WT, cv. Erythrospermum-35, with the greatest means in GWP and TGW (Table S1). The two studied mutant lines, M/1 and M/2, had significantly higher GWP by 2.0- and 2.1-fold, respectively, compared to the WT, with subsequent increased GWS in the M/2 by 1.8-fold. The M/2 mutant line had significantly higher GNS, GWP and TGW in the range of 1.4–1.5-fold compared to cv. Erythrospermum-35.

Additionally, yield-associated traits in WT and mutant lines, M/1 and M/2, were studied and the results revealed strong associations between the components of grain morphometry and Fe and Zn content in grains (Table S3). Specifically, grain Fe content in M/2 line showed positive and highly significant correlation with Zn content (r^2^ = 0.996*), and also GNS was correlated with GL (r^2^ = 0.998*), but TGW had a positive and significant correlation with GA (r^2^ = 0.996*) in the WT only.

### Expression of genes involved in phytosiderophore synthesis, secretion and expression and modulation of bHLH transcription factor in roots and leaves of mutant lines and WT

To evaluate the molecular response of plants in M_5_ mutant lines and in the WT bread wheat parent, cv. Erythrospermum-35, the expression of several groups of genes related to the different steps of Fe and Zn accumulation were assessed in root and leaf tissue samples. These studied genes were involved in phytosiderophore (PS) synthesis and secretion, long-distance Fe transport, intracellular Fe transport, storage proteins, and the regulation of TF bHLH.

**Figure 3:**
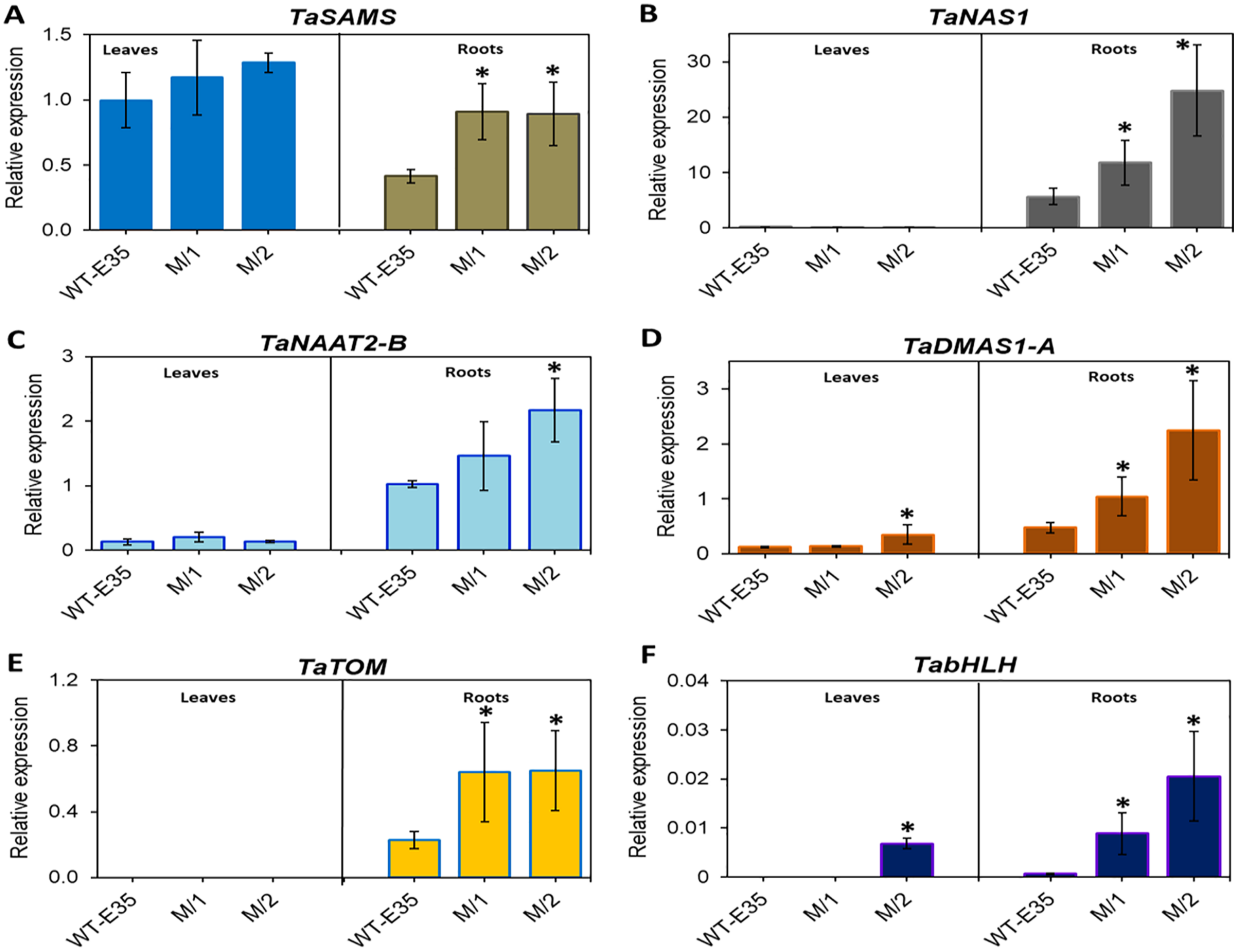
Differences in expression levels of wheat homologs of phytosiderophore synthesis-related genes and TF: (A) *TaSAMS*, S-adenosyl metioninesynthetase (Ta.69768); (B) *TaNAS1*, Nicotianamine synthase (Ta.37977); (C) *TaNAAT2-B*, Nicotianamineaminotransferase (Ta.4977); (D) *TaDMAS1-A*, Deoxymugineic acid synthase (Ta.5335); (E) *TaTOM*, Transporter of mugineic acid (Ta.5180), and (F) *TabHLH* transcription factor, in leaves and roots of WT cv. Erythrospermum-35, bread wheat parent, and in M5 mutant lines (M/1 and M/2). Expression data were normalized relative to the expression of the reference gene Ta.22845 (ATP-dependent 26S proteasome regulatory subunit). Values are the mean ± standard deviation of three biological replicates. Asterisks indicate the statistical difference between the parent and the mutant lines (*P < 0.05).

In leaves of the parental WT, relative mRNA levels of *TaSAMS* (Ta.69768) were 2.4-fold higher than those in roots, but in both mutant lines there were no differences in leaf and root expression. However, up-regulation of root specific TaSAMS in both mutant lines was two-fold and significant compared to the WT parent (Fig. 3A). Three other genes, which are also involved in PS biosynthesis, *TaNAS1* (Ta.37977) (Fig. 3B), *TaNAAT2-B* (Ta.4977) (Fig. 3C), and *TaDMAS1-A* (Ta.5335) (Fig. 3D), showed a very low level of expression in leaves for all wheat genotypes, with the exception of *TaDMAS1-A* in M/2 mutant line (Fig. 3D). In contrast, the expression of the same genes in roots was apparent in all wheat genotypes. However, expressions of *TaNAS1*, *TaNAAT2-B*, and *TaDMAS1-A* in roots of both mutant lines were significantly up-regulated by 2.1–4.7-fold, exceeding that of the WT parent. Only one mutant line, M/1, did not have a significant difference in *TaNAAT2-B* compared to the WT parent due to greater variability, but the trend for higher expression was clear (Fig. 3C). In particular, the most significant increase was found in *TaNAS1* and *TaDMAS1-A* expression in mutant line M/2 (4.4- and 4.7-fold, respectively, compared to that of WT, cv. Erythrospermum-35) (Figs. 3B and 3D). The *TaTOM* homolog Ta.5180 gene, encoding a PS efflux transporter, was only expressed in roots, with a strong tendency for increased expression in both mutant lines (Fig. 3E). Thus, the combined overexpression of five root-specific genes, *TaSAMS*, *TaNAS1*, *TaNAAT2-B*, *TaDMAS1-A* and *TaTOM*, in association with increased grain Fe/Zn content of both spring bread wheat M_5_ mutant lines were revealed, with expression levels significantly higher compared to the original WT parent (Fig. 3).

Additionally, the TF gene *TabHLH* homolog Ta.34545 was significantly up-regulated to a large degree in the roots of both M/1 and M/2 mutant lines (13.1- and 30.2-fold, respectively), and in leaves of M/2 mutant lines (Fig. 3F). No or poor expression of TF *TabHLH* in leaves and roots was found in the WT parent cv. Erythrospermum-35.

### Expression of genes encoding long-distance Fe transporters and intracellular Fe transporters and storage protein

**Figure 4:**
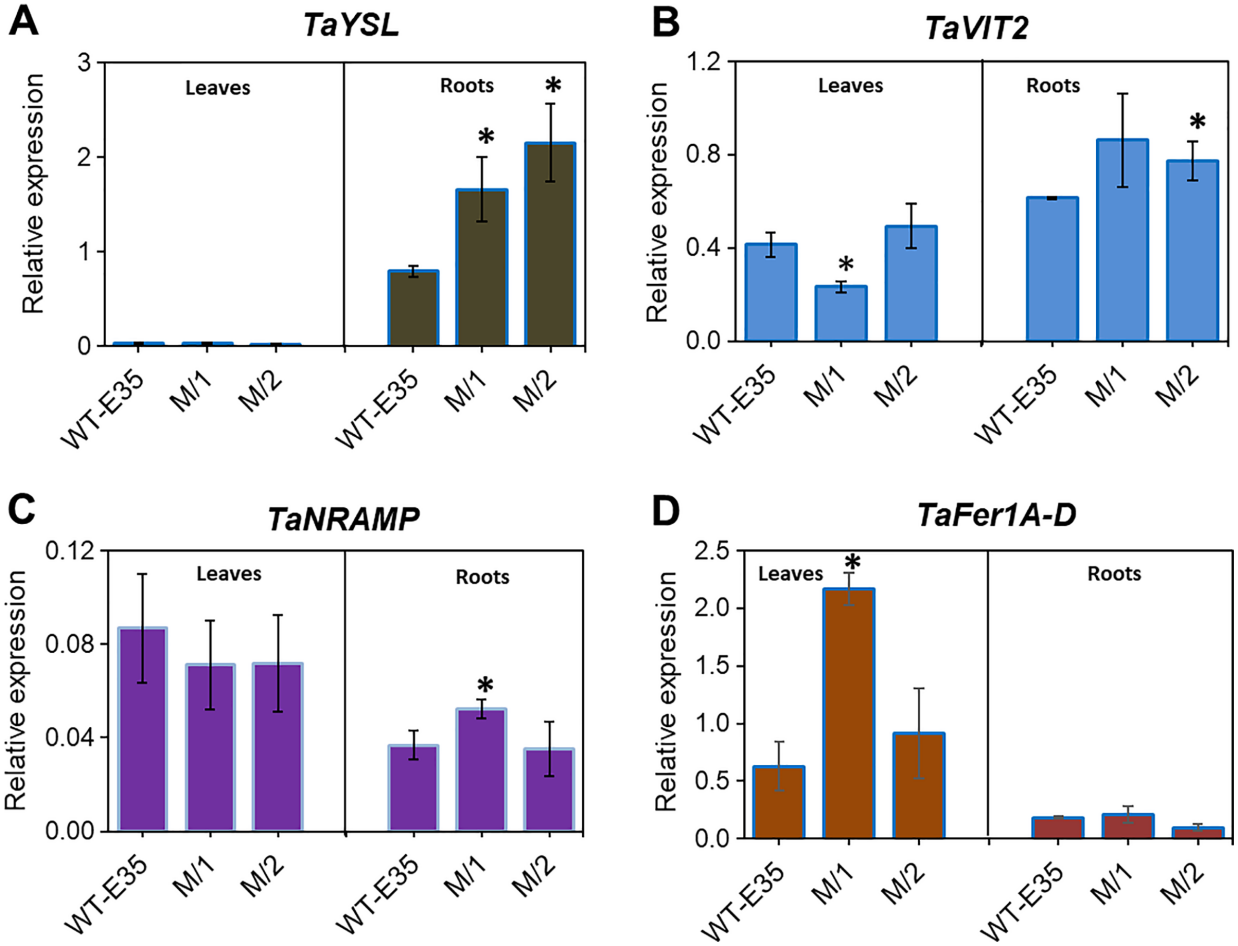
Differences in expression levels of wheat homologs for genes encoding long-distance Fe transporters, storage proteins and intracellular Fe transporters: (A) *TaYSL*, yellow-stripe-like protein; (B) *TaVIT2*, vacuolar iron transporter; (C) *TaNRAMP*, natural resistance-associated macrophage protein; and (D) *TaFer1-D*, Fe-regulated protein (ferritin), in leaves and roots of WT cv. Erythrospermum-35, bread wheat parent, and in M_5_ mutant lines (M/1 and M/2). Expression data were normalized relative to the expression of the reference gene Ta.22845 (ATP-dependent 26S proteasome regulatory subunit). Values are the mean ± standard deviation of three biological replicates. Asterisks indicate the statistical difference between the parent and the mutant lines (*P < 0.05).

Yellow-stripe-like proteins, encoded by *TaYSL* genes, are involved in long-distance Fe transport in roots and leaves of wheat plants and are related to grain Fe/Zn content. The expression patterns of *TaYSL* in WT spring wheat parent and mutant lines are shown in Fig. 4A. A significantly higher expression of root-specific *TaYSLA* (Ta.48303) was found in both mutant lines compared to the WT parent, cv. Erythrospermum-35. This gene encodes the wheat homologs of the metal-NA transporter YSL, and in addition to its transport role, it is also involved in PS synthesis and secretion. A very low expression level of *TaYSLA* was found in leaves, with no variation in its level among wheat genotypes (Fig. 4A).

Expression profiling of root and leaf tissues revealed differential expression of the vacuolar transporter *TaVIT2*, homologous group 2 (Ta.22757) (Fig. 4B). The organ-specific difference in expression was most observed in the M/1 mutant line because leaf *TaVIT2* was expressed at a significantly lower level compared to the WT parent and M/2 mutant line. However, the overall *TaVIT2* expression pattern in leaves suggests maintenance of their participation in long-distance Fe transport. A significant increase in *TaVIT2* expression was only observed in the roots of the M/2 mutant line while M/1 mutant line failed to reach significance due to high variability in the gene expression data. The *TaVIT2* expression profiles showed dependence on tissue specificity in spring wheat genotypes with different grain Fe/Zn contents. This may be linked to genotypic differences in metal homeostasis directing sink and source demands, as well as differences in remobilization during grain filling.

The *TaNRAMP* homolog (Ta.13247) encodes a natural resistance-associated macrophage protein that plays an important role as an intracellular Fe transporter. Tissue-specific expression of *TaNRAMP* in the WT parent and two mutant lines are shown in Fig. 4C, where expression was higher in total in leaves compared to roots. However, in the parent cultivar only, leaf *TaNRAMP* expression was significantly higher (2.4-fold) than that in roots. In roots, significantly higher expression (1.4-fold) of *TaNRAMP* was observed in the M/1 mutant line than in the WT parent (Fig. 4C). In our results studying the role of tissue specificity and genotypic variation in reference to grain Fe content, ferritin storage protein-related *TaFer1A-D* (Ta.5220) in wheat showed a predominance of leaf expression. There was a 3.5-fold increase in the M/1 mutant line compared to the WT parent, but it was not significant in M/2 mutant line (Fig. 4D).

Summarizing the presented results, higher grain Fe and Zn content in two new M_5_ mutant lines of spring bread wheat, developed through gamma-radiation treatment compared to plants of WT parent cv. Erythrospermum-35, was associated with the up-regulation of genes in roots and leaves, indicating the strong combined contribution of genes involved in phytosiderophore synthesis and secretion (*TaNAAT2-B*, *TaNAS1* and *TaDMAS1-A*), long-distance Fe transport (*TaYS1A*), and particularly, transcriptional regulation (*TabHLH*).

## Discussion

The present study showed distinct organ-specificity and genotype-dependent expression in a WT spring bread wheat parent and two M_5_ mutant lines derived from treatment with 200 Gy irradiation that had significantly higher grain Fe and Zn content than the WT by 1.6–1.7-fold and 1.6–2.1 times, respectively. PA content was lower by 1.8–2.8-fold, which provided 2.9–5.8 times higher metal bioavailability than that in the WT parent. Along with grain trace elements, the nutritional value of a wheat crop also depends on grain protein content (GPC) and on the protein composition, which greatly influences the kind of end products it may be used for (Balyan et al., 2013). Variation in GPC is limited in current commercial wheat cultivars and, therefore, the breeding program for improvement of GPC is difficult. The GPC in the mutant line M/2 was significantly higher by 10.3% compared to the WT parent. Wheat grain nutrient value is subject to the combined yield traits of grain weight, size and shape (Huang et al., 2015). Therefore, such morphological traits as GL, GW and A are important as phenotypically stable yield components (Gegas et al., 2010). Concerning the presented results, GL and GA were significantly increased in one and both mutant lines, respectively compared to the WT parent, but GW was not increased.

The partitioning of mineral elements (Fe and Zn) within cereal grains is affected by aspects of grain morphology, such as grain size, embryo size, and the number and thickness of the tissue layers (White & Broadley, 2009). It was shown that translocation of Zn from root to shoot following Zn uptake by roots is affected by many factors. For instance, the foliar application of Zn and Fe as a practical approach increased grains Zn and Fe content and improved their quality (Niyigaba et al., 2019). Additionally, the translocation of Zn and Fe from flag leaves to grains in wheat was also facilitated by metal-chelating compounds, such as 2-deoxymugineic acid (DMA) (Barunawati et al., 2013). It was also shown that sulfur presence in the growing medium in cereal crops enhanced the ability of plants to absorption and accumulates Fe (Zuchi, Cesco & Astolfi, 2012), particularly in Fe-deficient conditions (Wang, Kawakami & Bhullar, 2019). Our presented results show high accumulation of Fe in grains of two mutant lines, which is strongly associated with Fe translocation from roots to grains compared to WT cv. Erythrospermum-35 (Fig. 1).

The cereal grain consists of four major tissues as follows: embryo, aleurone, starchy endosperm and outer layers (testa and pericarp). Elemental microanalyses of wheat grain sections detected that phosphate, potassium, calcium, manganese, iron and zinc appear to be concentrated in a similar way, with the highest content being in the aleurone and the embryo (in particular the scutellum) and only a low level in the starchy endosperm (Brinch-Pedersen et al., 2007). Sulfur, copper and chloride, contrastingly, were fairly equally distributed between the different tissues. In our present study, the distribution of Fe in grains of WT and two M_5_ mutant lines, M/1 and M/2, stained using Perls’ Prussian blue, revealed visible positive blue staining in embryo, aleurone layer and slight staining in scutellum, but only rare and weak inclusions of Fe in the endosperm (Figs. 2A and 2B). In mutant lines with higher content of Fe than in WT (Table 1), the Fe staining was visibly increased and was distributed in the embryo, scutellum, strongly visible in the aleurone layer, and especially around the groove and in the endosperm patches compared to WT (Figs. 2C–2F). It has been reported that Fe staining was increased noticeably around the grain groove in wheat genotypes overexpressing *TaVIT2*, with weak portions of staining in the endosperm (Connorton et al., 2017). Fe levels were consistently enhanced 2-fold in white flour, from 9.7 mg/kg in control lines to 21.7 mg/kg in lines with a single overexpressing copy of *HMW*-*TaVIT2* (Connorton et al., 2017). Additional transgene copies resulted in a similar 2-fold increase in Fe, whereas lines with 20 or more copies had 4-fold higher Fe compared to controls, up-to 41.5 mg/kg (Connorton et al., 2017). Specific Prussian blue staining of Fe in transgenic indica rice (Krishnan et al., 2003) and red color Zn-DTZ complex staining in wheat (Ozturk et al., 2006) also illustrated that Fe and Zn were present in the highest content in the aleurone layer and the embryo.

Development of Fe-biofortified wheat lines was unsuccessful by traditional breeding methods because it was found that yield was negatively correlated to Fe content, and the research interest was focused on yield rather than for Fe content in past years (Connorton & Balk, 2019). Yield is unalterably the most significant goal characterizing new wheat genotypes and germplasms. Considering the yield associated traits analyzed in this study, such as GNS, GWS, GWP, and TGW, the presented results showed that wheat mutant lines significantly exceeded WT, cv. Erythrospermum-35, in these parameters with the greatest means in GWP and TGW (Table S1). The studied mutant lines, M/1 and M/2, showed significantly higher GWP by 2.0- and 2.1-fold, respectively, compared to WT, with subsequent GWS for the M/2 increased by 1.8-fold. The M/2 mutant line had significantly higher GNS, GWP and TGW in the range of 1.4–1.5-fold compared to cv. Erythrospermum-35.

Additionally, the significant associations between yield-associated traits, grain morphometry, and Fe and Zn content were found in WT and mutant lines, M/1 and M/2 (Table S3). The M/2 grain Fe content showed significant and high positive correlation with Zn content (r^2^ = 0.996*) and also GNS with GL (r^2^ = 0.998*), but the WT TGW significantly and positively correlated with GA (r^2^ = 0.996*).

The analysis of 10 selected genes revealed significant up-regulation, mainly in the root expression of key components involved in the Strategy II mode of Fe uptake, transport, and storage (Figs. 3 and 4, Table S4). The presented results show that in the studied mutant lines, the expression of genes related to PS synthesis, including *TaSAMS*, *TaNAS1*, *TaNAAT2-B*, *TaDMAS1-A*, and secretion-related *TaTOM* homologs were significantly higher in roots of both M/1 and M/2 compared to the WT parent, cv. Erythrospermum-35. The only exception was found in *TaNAAT2-B*, where root expression in the M/1 mutant line was not significant (Fig. 3C). In general, this finding suggests that these mutant lines are characterized by an increased production of NA through the participation of SAMS and NAS enzymes and subsequent reactions of DMA formation by DMAS and PS efflux *via* the involvement of the TOM transporter. These are vital for Fe ion acquisition and homeostasis (Nozoye et al., 2011; Kobayashi & Nishizawa, 2012). Comparison of fold differences revealed that there was genotypic variation in the level of root-specific expression of these genes between lines. For example, the M/2 mutant line was characterized by the highest level of expression of *TaNAS1* and *TaDMAS* (4.4- and 4.7-fold increase, respectively) compared to the WT parent (Figs. 3B and 3D). These results suggest the direct influence of higher gene expression related to PS synthesis on the metal content of xylem-fed organs (roots and leaves) in mutant lines.

In addition to increased PS production, in roots of the M/2 mutant line a 2.8-fold higher expression of *TaTOM* was observed. This gene encodes the transporter crucial for the secretion of PS into the soil. *TaTOM* is involved in DMA efflux into the rhizosphere and contributes to its internal transport, which is required for normal plant growth (Fig. 3E). It has been reported that expression of *TOM* genes in barley and maize, *HvTOM1* and *ZmTOM1*, were strongly induced in Fe-deficient roots. Additionally, *HvTOM1* and *OsTOM1* in barley and rice, respectively, showed a diurnal pattern of day/night cycling in their expression (Nozoye et al., 2011; Nozoye, Nakanishi & Nishizawa, 2013). Stronger combined up-regulation of *TaSAMS*, *TaNAS1*, *TaDMAS1-A* and *TaTOM* in the roots of both mutant lines and *TaNAAT2-B* in the M/2 line compared to the WT parent revealed that the studied mutant lines have the potential to increase PS synthesis and secretion. This also reflects the functioning of the basic components of the Strategy II mode for Fe/Zn ion acquisition and efflux. Such genotype-specific gene expression patterns were found to be correlated with high grain Fe and Zn content and consequently could attest to their biofortification capacity, identifying them as a novel valuable genetic resource to combat malnutrition. Improving the efficiency of Fe uptake in crops like bread wheat has a complex positive effect on crop productivity and human health.

Transcription factor bHLH is one of the most important classes of TFs that affect Fe homeostasis (Heim et al., 2003; Yang et al., 2016; Gao et al., 2019; Hao et al., 2021). The importance of bHLH is greatly enhanced under Fe-deficient growth of cereal plants, positively regulating responses by inducing *TabHLH* expression; as recently identified in rice (Kobayashi et al., 2019) and wheat (Wang, Kawakami & Bhullar, 2019). Their regulatory role includes genes involved in the synthesis of PSs, transporters, and other genes related to maintaining Fe homeostasis. In line with results presented in the current study, the highest expression was recorded in the roots of both mutant lines as 13.1- and 30.2-fold increases in *TabHLH* expression compared to the WT parent. In all wheat genotypes, *TabHLH* expression in leaves was very low or undetectable (Fig. 3F, Table S4). Notably, FIT, a bHLH transcription factor, is the chief regulator of Fe deficiency responses and homeostasis in Arabidopsis. The TabHLH polypeptide interacts with different proteins, controlling the expression of various genes involved in Fe uptake and homeostasis, and acting as a FIT-regulated network in Fe deficiency response (Wu & Ling, 2019). In keeping with this, high expression of *TabHLH* in roots of new spring wheat mutant lines strongly support the given statement. Additionally, during plant growth under Fe shortage, *TabHLH* was significantly up-regulated in shoots and roots of another wheat cv. Zhenis with corresponding M_5_ mutant line (Kenzhebayeva, Atabayeva & Sarsu, 2021). Thus, *TabHLH* could be a target gene for the biofortification of wheat.

The key role in long-distance Fe transport and its mobilization from the rhizosphere to the vegetative and reproductive parts of cereal plants belongs to the YSL family of transporters (Kumar et al., 2019; Gupta et al., 2021). In wheat, evidence of the YSL subfamily been published only recently (Kumar et al., 2019; Wang, Kawakami & Bhullar, 2019). It has been shown that putative YSL proteins have variations in their level of expression across tissues and stages of grain development (Kumar et al., 2019). Relative to the difference in grain Fe content, the expression of *TaYSL* genes in roots and leaves of wheat mutant lines in the present study revealed a significant 2.1- and 2.7-fold increase in roots (Fig. 4A, Table 1). In accordance with the increased expression of *TaYSL*, the up-regulation of *TaSAMS*, *TaNAS1*, *TaNAAT2-B*, and *TaDMAS1-A* were observed in the roots of mutant lines, indicating the importance of both Fe ion acquisition and subsequent transport in the leaf tissues, resulting in grain mineral biofortification. Stronger expression of *TaTOM* in the roots of the mutant lines compared to the WT parent may indicate for the involvement of the gene in long-distance transport from leaves to grain, thus contributing to enhanced grain Fe/Zn content, in addition to its role in metal uptake from the rhizosphere. Consistent with the results of high Fe translocation in wheat mutant lines (Fig. 1), the overexpression of some genes could indicate a relationship to xylem loading and unloading of metals and the important contribution of this step in the movement of Fe to grains. Identifying the genotype-dependent and root-specific differential expression of genes related to metal homeostasis in mutant lines of spring wheat varying in grain metal content is an important step in prioritizing genes for wheat biofortification.

Fe sequestration into vacuoles and ferritin complexes are two proposed major storage mechanisms in plants (Briat et al., 2010; Borg et al., 2012; Connorton et al., 2017; Connorton & Balk, 2019; Gupta et al., 2021). Analysis of *TaVIT2* expression in the root and leaf tissues of wheat mutant genotypes showed a slight up-regulation in the roots of both mutant lines with increased grain Fe/Zn content (Fig. 4B). The diverse role of the wheat VIT2 transporter in maintaining and governing Fe usage and storage, and therefore, re-distributing metal ions between tissues was demonstrated by the results of studies on the expression of the Vacuolar-iron transporter-like (*VTL*) genes (*TaVTL1*, *TaVTL2*, and *TaVTL4*) during Fe deprivation (Sharma et al., 2020).

NRAMPs, which represent plasma membrane-localized proton/metal transporters, are involved in metal homeostasis, and constitute a component of their transport to the shoots and leaves. Due to the exceptional significance of NRAMPs, the corresponding genes have been characterized in various plant species (Tejada-Jiménez et al., 2015; Qin et al., 2017; Gao et al., 2018; Gupta et al., 2021), including a study on bread wheat (Wang, Kawakami & Bhullar, 2019). Our presented results indicate that *TaNRAMP* was expressed in leaf and root tissues, with a slight predominance in leaves (Fig. 4C). The genotype-dependent pattern was manifested in significantly increased *TaNRAMP* expression in roots of the M/1 mutant line (1.4-fold). Such expression could imply their role in wheat biofortification, but to a lesser extent compared to the potential of genes involved in Fe uptake and/or transport. However, the importance of TaNRAMPs in wheat has been shown in experiments in which high expression of *TaNRAMP* was induced by Fe starvation (Kaur et al., 2019; Wang, Kawakami & Bhullar, 2019).

Considering the role of the second storage mechanism in plants, Fe sequestration into ferritin, and the genotype-specific expression of Fe homeostasis-related genes, the data of *TaFer1A-D* expression show its strong leaf-specific expression, while the M/1 mutant line had significantly increased *TaFer1A-D* expression compared to the WT parent (3.5-fold) (Fig. 4D, Table S4). This response suggests the ability of the mutant line to sequester metal in a sink-driven manner to support biofortification.

## Conclusions

The present study identified the genotype-dependent and organ-specific differential expression of genes involved in Fe homeostasis in spring bread wheat genotypes differing in grain Fe and Zn content, PA content, and metal bioavailability. Mutant lines generated in the background of WT cv. Erythrospermum-35, typified by their strong improvement in metal biofortification ability, showed a significantly higher level of root-specific gene expression related to the synthesis of Fe chelators (NA and DMA) and genes encoding various Fe transporters. Fe homeostasis is seemingly governed by the broad modulation of various genes and gene families in wheat mutant lines. Regarding the high grain Fe and Zn content, overall, Fe uptake and transport were better regulated by roots, being the first step of metal ion acquisition and with the strong contribution of TFs, such as the TabHLH family. Two new mutant genotypes of spring bread wheat were identified in the present study along with target genes for Fe/Zn biofortification, thus widening our understanding of the role of metal homeostasis-related genes in Fe/Zn uptake, transport, and redistribution in wheat grains.

## Supplemental Information

10.7717/peerj.13515/supp-1 Supplemental Information 1 Mean yield-associated traits of spring wheat M_5_ M/1 (144/1) and M/2 (153/5) mutant lines originating from 200 Gy treatment of the parent (WT) cv. Erythrospermum-35′. Grain number and weight per main spike, grain weight per plant are the mean of five replicates of randomly selected spikes/plants. Asterisks indicate significance at *P, 0.05, **P, 0.01 and ***P, 0.001

10.7717/peerj.13515/supp-2 Supplemental Information 2 Information about primers used for qPCR in this study, including NCBI GenBank ID and Unigene ID (in brackets), primer sequence, amplicon size and primer efficiency.

10.7717/peerj.13515/supp-3 Supplemental Information 3 Coefficients of correlation (r^2^) between grain Fe and Zn content, grain protein content (GPC), grain morphometric parameters: grain area (GA), grain length (GL), and grain width (GW) with yield-associated traits. Grain number per main spike (GNS), grain weight per main spike (GWS), grain weight per plant (GWP), and 1,000 grain weight (TGW) in WT (spring wheat cv. Erythrospermum-35) and mutant lines M/1 (144/1) and M/2 (153/5).

10.7717/peerj.13515/supp-4 Supplemental Information 4 Gene expression fold changes in roots and leaves and between plants of WT parent, spring wheat cv. Erythrospermum-35 and mutant lines, M/1 and M/2. ‘-’, no significant difference between gene expression level and among WT and mutant lines; 0, the gene was not expressed; value in parentheses – level of gene expression, obtained from the calculation; values on the table represent fold changes, observed in plants of mutant lines as compared to WT (statistically significant changes); ↑, ↓ – up-and down-regulation for a particular gene relative to the parent.

## Additional Information and Declarations

### Data Availability

The following information was supplied regarding data availability: The raw measurements are available in the Supplemental Files.
