# Supplementary material for: Organ-specific expression of genes involved in iron homeostasis in wheat mutant lines with increased grain iron and zinc content
Source: PeerJ. 2022 Jun 10;10:e13515. doi: 10.7717/peerj.13515 (PMC9190668; doi:10.7717/peerj.13515)
Supplement: Supplemental Information 1 — Grain number and weight per main spike, grain weight per plant are the mean of five replicates of randomly selected spikes/plants. Asterisks indicate significance at *P, 0.05, **P, 0.01 and ***P, 0.001 [file peerj-10-13515-s001.docx]

**Supplementary Materials**

Kenzhebayeva S., Atabayeva Saule, Sarsu F., Abekova A., Shoinbekova S., Omirbekova N., Doktyrbay G., Beisenova A., Y. Shavrukov. Organ-specific expression of genes involved in iron homeostasis in wheat mutant lines with increased grain iron and zinc content

**Supplementary Table S1.** Mean yield-associated traits of spring wheat M_5_ M/1 (144/1) and M/2 (153/5) mutant lines originating from 200 Gy treatment of the parent (WT) cv. Erythrospermum-35’. Grain number and weight per main spike, grain weight per plant are the mean of five replicates of randomly selected spikes/plants. Asterisks indicate significance at *P, 0.05, **P, 0.01 and ***P, 0.001

| Genotypes | Grain number per main spike  (GNS) | Grain weight per main spike  GWS (g) | Grain weight per plant  GWP (g) | Thousand grain weight  TGW (g) |
| --- | --- | --- | --- | --- |
| cv. Eritrospermum-35 | 30.33±2.08 | 1.60±0.19 | 2.13±0.39 | 34.13±0.08 |
| M/1 (144/1) mutant line | 44.67±3.22** | 2.25±0.15* | 4.31±1.23* | 48.48±8.36* |
| M/2 (153/5) mutant line | 58.67±9.02** | 2.87±0.38** | 4.43±0.87* | 40.54±3.60* |

.
