# Supplementary material for: Organ-specific expression of genes involved in iron homeostasis in wheat mutant lines with increased grain iron and zinc content
Source: PeerJ. 2022 Jun 10;10:e13515. doi: 10.7717/peerj.13515 (PMC9190668; doi:10.7717/peerj.13515)
Supplement: Supplemental Information 2 [file peerj-10-13515-s002.docx]

**Supplementary Materials**

Kenzhebayeva S., Atabayeva Saule, Sarsu F., Abekova A., Shoinbekova S., Omirbekova N., Doktyrbay G., Beisenova A., Y. Shavrukov. Organ-specific expression of genes involved in iron homeostasis in wheat mutant lines with increased grain iron and zinc content

.

**Supplementary Table S2.** Information about primers used for qPCR in this study, including NCBI GenBank ID and Unigene ID (in brackets), primer sequence, amplicon size and primer efficiency.

| **Gene name** | **NCBI accession** | **F and R** | **Sequence (5’-3’)** | **Ampli-con size (bp)** | **Primer effici-ency** |
| --- | --- | --- | --- | --- | --- |
| *TaSAMS* | HP612105 (Ta.69768) | F | GCGCACGATCTCTCGTAGT | 61 | 2.00 |
|  |  | R | GTCATGGTCTTTGGCGAG |  |  |
| *TaNAS1* | JP215700.1 (Ta.37977) | F | aggcgcactactccgaca | 61 | 1.83 |
|  |  | R | gaagatgccgaggtggtc |  |  |
| *TaNAAT2-B* | BT009504 (Ta.4977) | F | GACCATTTAGCCAAGGTTGC | 64 | 1.85 |
|  |  | R | TACCTCGTCAGCAATCACCA |  |  |
| *TaDMAS1-A* | AB269908 (Ta.5335) | F | CACCGTCAATCAGGTGGAG | 68 | 1.88 |
|  |  | R | CCTCTGCAGAACTCCCTCA |  |  |
| *TaTOM* | JP874085 (Ta.5180) | F | TGGAGAATGCAATGATAGGTTTT | 72 | 1.86 |
|  |  | R | AGATGTTTTGCCTCGCTGTT |  |  |
| *TabHLH* | CD872522 (Ta.34545) | F | GGCTAGGTAGCTACGTTCCATC | 51 | 2.00 |
|  |  | R | TGATCCATCACAGGCAGTTG |  |  |
| *TaYSLA* | HP631418 (Ta.48303) | F | TGCATGGAACCAAGATAAACAAG | 68 | 2.00 |
|  |  | R | ACATATCAAAGCGGATGCAA |  |  |
| *TaVIT2-D* | BE426855 (Ta.22757) | F | GGCCTCGGAGGGTATCTG | 64 | 1.86 |
|  |  | R | ACAGTATGTCCGCGATCTCC |  |  |
| *TaNRAMP* | AK334756 (Ta.13247) | F | TGGAAAGGAGCTTCTGATCG | 51 | 2.00 |
|  |  | R | GCCTGCTTGATGGTCTTTG |  |  |
| *TaFer1A-D* | HP635522 (Ta.5220) | F | GAGTGTGGCACTTCGATCAG | 61 | 2.00 |
|  |  | R | CGTAGTCGTCGTCGTGTCCTTCA |  |  |
| *ATP-dep. 26S proteasome regul. subunit* | TC353778 (Ta.22845) | F | GCTGGCTCGTTCAACTGATG | 202 | 1.87 |
|  |  | R | GGACCAAGCGTTCTGATTACTC |  |  |
