# Supplementary material for: Organ-specific expression of genes involved in iron homeostasis in wheat mutant lines with increased grain iron and zinc content
Source: PeerJ. 2022 Jun 10;10:e13515. doi: 10.7717/peerj.13515 (PMC9190668; doi:10.7717/peerj.13515)
Supplement: Supplemental Information 3 — Grain number per main spike (GNS), grain weight per main spike (GWS), grain weight per plant (GWP), and 1,000 grain weight (TGW) in WT (spring wheat cv. Erythrospermum-35) and mutant lines M/1 (144/1) and M/2 (153/5). [file peerj-10-13515-s003.docx]

**Supplementary Materials**

Kenzhebayeva S., Atabayeva Saule, Sarsu F., Abekova A., Shoinbekova S., Omirbekova N., Doktyrbay G., Beisenova A., Y. Shavrukov. Organ-specific expression of genes involved in iron homeostasis in wheat mutant lines with increased grain iron and zinc content

**Supplementary Table S3.** Coefficients of correlation (r^2^) between grain Fe and Zn content, grain protein content (GPC), grain morphometric parameters: grain area (GA), grain length (GL), and grain width (GW) with yield-associated traits: grain number per main spike (GNS), grain weight per main spike (GWS), grain weight per plant (GWP), and 1000 grain weight (TGW) in WT (spring wheat cv. Erythrospermum-35) and mutant lines M/1 (144/1) and M/2 (153/5).

| **Genotype** | **Trait** | **Zn content (mg/kg)** | **Fe content (mg/kg)** | **GPC, %** | **GA (mm^2^)** | **GL (mm)** | **GW (mm)** |
| --- | --- | --- | --- | --- | --- | --- | --- |
| WT, cv. Erythrospermum-35 | Fe content | 0.914 | − | 0.408 | 0.761 | 0.032 | 0.969 |
| Mutant line M/1 (144/1) |  | 0.0317 | − | 0.089 | 0.271 | 0.600 | 0.269 |
| Mutant line M/2 (153/5) |  | 0.996* | − | 0.25 | 0.268 | 0.269 | 0.010 |
| WT, cv. Erythrospermum-35 | Zn content | − | − | 0.699 | 0.956 | 0.212 | 0.328 |
| Mutant line M/1 (144/1) |  | − | − | 0.785 | 0.871 | 0.235 | 0.384 |
| Mutant line M/2 (153/5) |  | − | − | 0.309 | 0.328 | 0.871 | 0.0012 |
| WT, cv. Erythrospermum-35 | GNS | 0.012 | 0.158 |  | 0.010 | 0.691 | 0.306 |
| Mutant line M/1 (144/1) |  | 0646 | 0.197 |  | 0.286 | 0.829 | 0.286 |
| Mutant line M/2 (153/5) |  | 0.427 | 0.492 |  | 0.061 | 0.998* | 0.608 |
| WT, cv. Erythrospermum-35 | GWS | 0.634 | 0.881 |  | 0.423 | 0.0291 | 0.970 |
| Mutant line M/1 (144/1) |  | 0.859 | 0.0418 |  | 0.532 | 0.605 | 0.533 |
| Mutant line M/2 (153/5) |  | 0.154 | 0.204 |  | 0.010 | 0.932 | 0.871 |
| WT, cv. Erythrospermum-35 | GWP | 0.260 | 0.547 |  | 0.100 | 0.280 | 0.718 |
| Mutant line M/1 (144/1) |  | 0.692 | 0.0158 |  | 0.332 | 0.790 | 0.333 |
| Mutant line M/2 (153/5) |  | 0.888 | 0.055 |  | 0.070 | 0.717 | 0.135 |
| WT, cv. Erythrospermum-35 | TGW | 0.944 | 0.739 |  | 0.996* | 0.431 | 0.571 |
| Mutant line M/1 (144/1) |  | 0.123 | 0.968 |  | 0.441 | 0.421 | 0.441 |
| Mutant line M/2 (153/5) |  | 0.029 | 0.926 |  | 0.505 | 0.772 | 0.982 |
