# Supplementary material for: Organ-specific expression of genes involved in iron homeostasis in wheat mutant lines with increased grain iron and zinc content
Source: PeerJ. 2022 Jun 10;10:e13515. doi: 10.7717/peerj.13515 (PMC9190668; doi:10.7717/peerj.13515)
Supplement: Supplemental Information 4 — ‘-’, no significant difference between gene expression level and among WT and mutant lines; 0, the gene was not expressed; value in parentheses – level of gene expression, obtained from the calculation; values on the table represent fold changes, observed in plants of mutant lines as compared to WT (statistically significant changes); ↑, ↓ – up-and down-regulation for a particular gene relative to the parent. [file peerj-10-13515-s004.docx]

**Supplementary Materials**

Kenzhebayeva S., Atabayeva Saule, Sarsu F., Abekova A., Shoinbekova S., Omirbekova N., Doktyrbay G., Beisenova A., Y. Shavrukov. Increased Iron and Zinc bioavailability in grain of wheat mutant lines is associated with organ-specific expression of genes involved in iron homeostasis.

**Supplementary Table S4.** Gene expression fold changes in roots and leaves and between plants of WT parent, spring wheat cv. Erythrospermum-35 and mutant lines, M/1 and M/2. ‘-‘, no significant difference between gene expression level and among WT and mutant lines; 0, the gene was not expressed; value in parentheses – level of gene expression, obtained from the calculation; values on the Table represent fold changes, observed in plants of mutant lines as compared to WT (statistically significant changes); ↑, ↓ – up- and down-regulation for a particular gene relative to the parent.

| **Genes** | **WT. Erythro- spermum-35** | **M/1 mutant line** | **Ratio M/1 mutant line / WT** | | **M/2 mutant line** | **Ratio M/2 mutant line / WT** | |
| --- | --- | --- | --- | --- | --- | --- | --- |
|  | Roots / Leaves | Roots / Leaves | Roots / Leaves | | Roots/ Leaves | Roots / Leaves | |
| **Phytosiderophore synthesis and secretion** | | | | | | | |
| *TaSAMS* | 0.42 | − | 2.20**↑** | − | − | 2.15**↑**- | *−* |
| *TaNAAT2-B* | 7.59 | 7.17 | − | − | 15.56 | 2.12**↑**- | *−* |
| *TaNAS1* | 28.16 | 86.76 | 2.08↑ | − | 161.44 | 4.38↑ | *−* |
| *TaDMAS1-A* | 3.90 | 7.73 | 2.18↑ | − | 6.40 | 4.70↑ | *−* |
| *TaTOM* | 0.23/0 | 0.64/0 | 2.79↑ | − | 0/0.65 | 2.84↑ | *−* |
| **Transcriptional regulation** | | | | | | | |
| *TabHLH* | 0.0007/0 | 0.009/0 | 13.05↑ | 0/0 | 3.0 | 30.21↑ | 0.007/0 |
| **Long-distance Fe transport** | | | | | | | |
| *TaYS1A* | 28.28 | 91.76 | 2.09↑ | − | 101.64 | 2.72↑ | − |
| *TaVIT2* | 1.48 | 3.68 | 0.57↓ | 1.41↑ | 1.57 | 1.26↑ | − |
| **Intracellular Fe transport and storage** | | | | | | | |
| *TaNRAMP* | 0.42 | − | − | − | − | − | − |
| *TaFer1A-D* | 0.54 | 0.09 | − | 3.46↑ | 0.12 | − | − |
